# Simultaneous laparoscopic bilateral nephrectomy in polycystic kıdney disease using same trochars placed in the midline

**DOI:** 10.1590/S1677-5538.IBJU.2019.0629

**Published:** 2020-09-02

**Authors:** Necmettin Penbegul

**Affiliations:** 1 Department of Urology Yalova Atakent Hospital Yalova Turkey Department of Urology, Yalova Atakent Hospital, Yalova, Turkey

## INTRODUCTION

To present Simultaneous Laparoscopic Bilateral Nephrectomy in massive renal sized autosomal dominant polycystic kidney disease (ADPKD) using midline trochar approach. According to the literature, our technique has provided use of least trochar number during this surgery.

## PRESENTATION

The patient was a 59-year-old female with a history of endstage renal disease requiring dialysis due to dominant polycystic kidney disease. We planned to perform bilateral nephrectomy due to the right renal mass. The patient was placed in the right lateral decubitus position for a left nephrectomy under general anesthesia. On the right lateral decubitus position 3 trochars were used. One umblical and 2 paraumblical trochars which were 5cm far from umblicus were located in the midline. Left nephrectomy was completed and left kidney was left inside the abdomen. Then the patient was repositioned to the left lateral decubitus position for right nephrectomy. Same 3 trochars were used but additionally a trochar was added for liver retraction during right nephrectomy. After right nephrectomy was completed a midline incision was made just below the umbilicus between two trochars. Both surgical specimens were then removed through this incision. Abdominal wall incision was closed using standard procedure. Drain catheter was performed through the supraumblical trochar site.

## RESULTS

Bilateral laparoscopic nephrectomy was successfully completed without surgical complications by using a total of 4 trochars. Three of these trochars were the same used for both kidney surgeries. Postoperatively, the patient was discharged without any complications. Pathology of the renal mass was clear cell renal cancer (pT2aNxMx).

## CONCLUSION

Our technique showed that the placement of the trochars in the midline allows for Simultaneous Laparoscopic Bilateral Nephrectomy in autosomal dominant polycystic kidney disease by same trochars and removal of the kidneys from the same incision. This technique leads to fewer trochars, single incision and superior cosmesis.

